# Development of social learning and play in BaYaka hunter-gatherers of Congo

**DOI:** 10.1038/s41598-019-47515-8

**Published:** 2019-07-31

**Authors:** Gul Deniz Salali, Nikhil Chaudhary, Jairo Bouer, James Thompson, Lucio Vinicius, Andrea Bamberg Migliano

**Affiliations:** 1 Department of Anthropology, University College London, London, WC1H 0BW United Kingdom; 2 Leverhulme Centre for Human Evolutionary Studies, Department of Archaeology, University of Cambridge, Cambridge, CB2 1QH United Kingdom; 3 Department of Anthropology, University of Zurich, 8057 Zürich, Switzerland

**Keywords:** Human behaviour, Behavioural ecology, Biological anthropology, Cultural evolution

## Abstract

High-fidelity transmission of information through imitation and teaching has been proposed as necessary for cumulative cultural evolution. Yet, it is unclear when and for which knowledge domains children employ different social learning processes. This paper explores the development of social learning processes and play in BaYaka hunter-gatherer children by analysing video recordings and time budgets of children from early infancy to adolescence. From infancy to early childhood, hunter-gatherer children learn mainly by imitating and observing others’ activities. From early childhood, learning occurs mainly in playgroups and through practice. Throughout childhood boys engage in play more often than girls whereas girls start foraging wild plants from early childhood and spend more time in domestic activities and childcare. Sex differences in play reflect the emergence of sexual division of labour and the play-work transition occurring earlier for girls. Consistent with theoretical models, teaching occurs for skills/knowledge that cannot be transmitted with high fidelity through other social learning processes such as the acquisition of abstract information e.g. social norms. Whereas, observational and imitative learning occur for the transmission of visually transparent skills such as tool use, foraging, and cooking. These results suggest that coevolutionary relationships between human sociality, language and teaching have likely been fundamental in the emergence of human cumulative culture.

## Introduction

The length of human childhood is an evolutionary puzzle. An average hunter-gatherer male spends the first 18 years of his life being dependent on others for food^1^. In contrast, chimpanzees are nutritionally self-sufficient immediately after weaning, at 5–6 years of age^1,2^. According to embodied capital theory, human childhood has evolved to allow necessary time to develop complex skills needed for the hunting and gathering niche^1^. Thus, there has been a growing interest in understanding how extant hunter-gatherer children learn skills and knowledge^3–5^. Nevertheless, there are a few gaps in the current literature on hunter-gatherer social learning that need to be addressed: (1) quantitative analyses of how reliance on different types of social learning varies across infancy, childhood and adolescence (2) whether social learning processes, such as teaching and imitation, are activity (domain) specific, (3) the relationship between play and social learning, how much time girls and boys spend in play throughout childhood, and at what age the play-work transition occurs. This paper aims to fill these gaps by examining the ontogeny of social learning processes and play in BaYaka hunter-gatherer children from Congo-Brazzaville by analysing video recordings and activity time budgets of children, ranging in age from early infancy to late adolescence.

Whilst culture, which we define here as socially learned behaviours, occurs in numerous non-human animals, many anthropologists and biologists argue that human culture is unique in being cumulative. Human cultural traits increase in diversity and complexity over time resulting in phenomena that no one individual could invent alone^6,7^. High-fidelity transmission is necessary for cumulative culture as it ensures cultural traits exist in a population long enough to be modified or combined to produce new variants and traits^7–9^. The human tendency to imitate and teach along with our cognitive capacity for language have been instrumental in the emergence of cumulative culture since they facilitate higher fidelity in transmission than other social learning mechanisms such as emulation^7,10^.

Humans’ reliance on imitation is so strong that in laboratory experiments, participants “over-imitate” actions of a model that are completely unnecessary for the achievement of their end goal^11,12^, and children imitate an actor’s actions even when they violate their society’s fairness norms^13^. Although there is substantial evidence of children’s reliance on imitation in laboratory experiments^14–16^, there are very few systematic observations of imitative learning in naturalistic settings^4^. These systematic observations on indigenous communities across Americas and Africa emphasize the role of children’s learning by observing, participating in and imitating the everyday activities of their community^4,17^. Nevertheless, there is no clarity regarding at what ages and for which activities children rely on imitative learning as opposed to other learning processes.

Another form of social learning that has been argued to contribute to high fidelity transmission of information is being taught^18^. There has been a debate as to the universality of teaching as some researchers working in small-scale societies have claimed teaching to be absent^19–22^. On the other hand, evolutionary theorists expect teaching to be universal as they argue teaching to be necessary for the transmission of complex information generated through cumulative cultural evolution^18^. In evolutionary biology teaching refers to instances when a knowledgeable individual modifies their own behaviour in the presence of a naïve individual, either at a cost to itself or with no immediate benefit, and as a result the naïve individual acquires new knowledge or skill earlier or quicker than s/he would have done otherwise^23^. Following this definition, scholars have documented cases of teaching in extant hunter-gatherers^20,24,25^, as well as reliance on active teaching for the acquisition of social norms^5^. Nevertheless, observations in hunter-gatherers indicate teaching to be rare and subtler than in Western societies^20,26,27^. Since hunter-gatherers value individual autonomy, adults are less likely to intervene in children’s actions and give direct instructions^4^. A seemingly contradictory aspect of a hunter-gatherer society is the dual emphasis on individual autonomy and cooperation. This “cooperative autonomy” is thought to contribute to a very different form of teaching than the Western one^24,28^. Unlike Western teaching, children are not obliged to conform to others’ requests and there is not a hierarchical relationship between the teacher and the learner^24^. Foragers are also expected to promote autonomy in their childrearing since self-reliance is crucial in an environment where a person needs to look for food each day^29^. If teaching co-evolved with cumulative culture to facilitate the transmission of complex information, then teaching should occur only for those activities that would be difficult to learn without direct guidance and instruction, which we aim to test here.

We also examine whether certain learning mechanisms are specific to certain activity and knowledge domains? Previously, we showed that among the BaYaka, plant knowledge is transmitted within families or the wider camp depending on its domain. While knowledge of medicinal use is shared within families, plant use related to foraging or social norms is shared with the wider camp^30^. Similarly, understanding for which activity domains specific social learning processes are employed may have added implications for our understanding of cultural transmission dynamics, and inform us regarding the evolutionary relationship between cumulative culture, teaching and language. For example, Fijian villagers employ teaching for high-skill and highly valued domains and parents are more likely to teach than any other kin^31^. Reviews on hunter-gatherer social learning suggest that teaching mostly occurs in domains that are difficult to learn such as ritual knowledge and skills related to social life such as sharing and cooperation^26^. Based on these observations we predict to observe teaching occurring for those knowledge domains that are opaque to the learner- that cannot be learnt by merely observing others. We predict observational and imitative learning, on the other hand, occurring for domains that can be readily acquired through watching others do. These forms of learning may especially be utilized during early childhood as they do not require verbal communication. Extant hunter-gatherers are good models to test these predictions and untangle the evolutionary relationships between teaching, culture and language because their way of life, characterized by small nomadic groups, lack of political hierarchies and food storage, mimic the environment in which human culture and cooperation have evolved. Thus, the skills and knowledge that are transmitted in extant hunter-gatherers are likely to resemble those learned in ancestral societies.

The final subject of this study relates to hunter-gatherer play. Playgroups provide a context where social learning can occur and many anthropologists have highlighted the importance of playgroups in hunter-gatherer childhood^4,32–34^. Building on the embodied capital theory^1^, some researchers have argued that play is an adaptation for learning and suggested that play activities should concern developing skills to the level of adult competency^34,35^. If play is an adaptation for learning skills, then we expect play activities to reflect the sexual division of labour in hunter-gatherers. As such, hunter-gatherer girls’ play activities would involve food gathering and domestic skills, whilst boys’ would relate to skills required for hunting and climbing trees for honey collection. Previous studies have supported the importance of play in skill acquisition^34,35^ and the presence of sex biases in play^34,36^. By analysing the work-themed play activities where children imitate work tasks in their play, researchers found that Aka hunter-gatherer girls spent more time in playing at food preparation than the Aka boys^34^. Aka boys, on the other hand, spent more time in playing at hunting, and honey collecting^34^. Analysing the ontogeny of play activities can inform us when the sexual division of labour emerges and how time allocated to work versus play changes in girls and boys. If play is an adaptation for learning skills, the relative time allocated to play versus subsistence effort will decrease as children approach adult levels of competence^35^. Only a few studies thus far have examined the ontogeny of sexual division of labour in hunter-gatherers. These studies have shown that a sexual division of labour emerges from middle childhood^37^ and girls decrease time spent in play at a greater rate than boys^34,36^.

Not only the activities of play but also the composition of playgroups can facilitate the acquisition of skills and knowledge necessary for the adult life. Hunter-gatherer playgroups are often composed of mixed-aged and mixed-sex children, and horizontal transmission among playmates is common^4,32,33^. For example, in a mixed-aged playgroup a younger child can be encouraged to share a food or play item by an older child. BaYaka hunter-gatherer children imitate adults’ forest spirit rituals in their ritual play (*mokondi massana*)^38^. During these rituals, women sing together polyphonically whilst hand-clapping to beckon the forest spirits into the camp and men prepare the forest spirits in a secret path, who later arrive in the camp and perform ritualistic dance. By imitating these rituals in mixed-sex playgroups, BaYaka children learn gender roles and cultural practices^38^. Hence play can facilitate the acquisition of subsistence skills via work themed play; social norms via supervision of younger members of the playgroup; and cultural values during other forms of play.

In this paper, we investigate the role and development of different social learning mechanisms and play in the acquisition of various skill and knowledge domains. We predict:

1. Observational and imitative learning occur in infancy and decrease after weaning as children become more efficient and start practicing skills with less need for a model.
2. Teaching occurs for the transmission of abstract information that cannot be transmitted via other social learning processes, whereas observational and imitative learning occur for visually transparent tasks such as using tools or food processing.
3. Playgroups provide the context for children to learn from each other by observing, imitating, play-practicing and, perhaps, teaching. We expect time allocated to play to increase after weaning (as dependence on mothers is reduced) and decrease throughout adolescence as skill proficiency increases, facilitating the transition from play to work. We expect the sexual division of labour to be reflected in children’s play activities.

## Results

### Learning processes change with age in hunter-gatherer children

**Figure 1:**
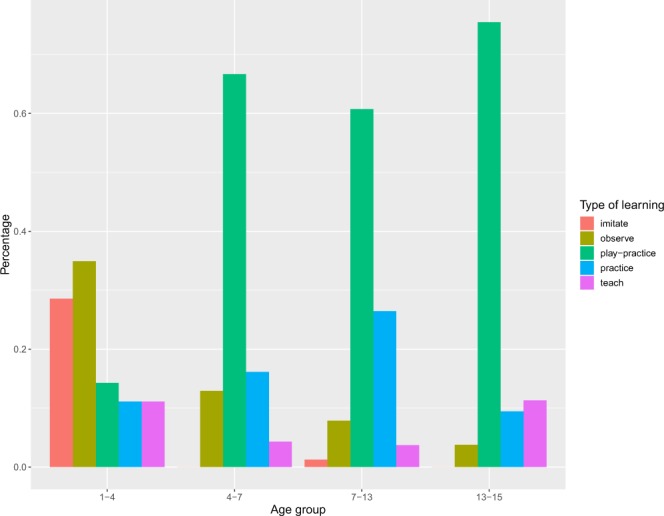
Percentage of number of entries for each learning process during infancy to early childhood (1–4 years), early childhood (4–7 years), middle childhood (7–13 years) and adolescence.

We observed that children younger than 4 years-old in our sample learnt mainly by imitation and observing. As children grew older learning through imitating and observing declined and the time spent in play increased (Fig. 1). The median ages of children in each entry where imitative learning or observation were recorded were 1.1 years and 5 years respectively. 86% of the imitative learning occurred in children aged below 4 years (Fig. 1). According to our regression model, the odds of imitating a model and observational learning declined by 47% and 30% for each year of age respectively (Fig. 1, Table 1).

**Table 1:** Mixed-effects logistic regression models on the odds of children learning through a specific learning process.

	Model 1: imitate		Model 2: observe		Model 3: practice		Model 4: teach		Model 5: play-practice	
	Odds ratio (CI lower, CI upper)	P-value	Odds ratio (CI lower, CI upper)	P-value	Odds ratio (CI lower, CI upper)	P-value	Odds ratio (CI lower, CI upper)	P-value	Odds ratio (CI lower, CI upper)	P-value
Age (1-year increase)	0.53 (0.34, 0.84)	<0.01	0.7 (0.57, 0.88)	<0.01	1.03 (0.88, 1.2)	0.73	0.85 (0.63, 1.17)	0.32	1.42 (1.12, 1.8)	<0.01
Sex (male)	0.39 (0.02, 6.94)	0.52	0.21 (0.04, 1.26)	0.09	0.31 (0.09, 1.09)	0.07	3.25 (0.29, 36.06)	0.34	5.73 (0.91, 35.97)	0.06
AIC	111.8		284.1		428.5		183		503.3	
Log likelihood	−51.9		−138.0		−210.2		−87.5		−247.6	

Odds of children using imitative learning (model 1), observational learning (model 2), practicing a skill (model 3), being taught (model 4), and play-practicing (model 5) with respect to sex and age. All models control for the focal child’s ID. *N* = 449 entries for 34 focal children. Data on 6 participants were not included in the models because we did not have age estimates for those participants.

Age did not affect the odds of learning skills through practice (Fig. 1, Table 1). Girls comprised almost 70% of the practice events. This is because the majority of these events concerned foraging related activities that the BaYaka women do. We will further analyse the activity-specificity of learning processes in the next section. Receipt of teaching was rare and accounted for 5.6% of all the entries and was not significantly predicted by age (Fig. 1, Table 1). Conversely, we observed a 42% increase in the odds of play-practice for each year increase in age (Table 1). Play-practice accounted for over 60% of childhood learning in our video sample (Fig. 1).

### Social learning processes are domain specific

**Figure 2:**
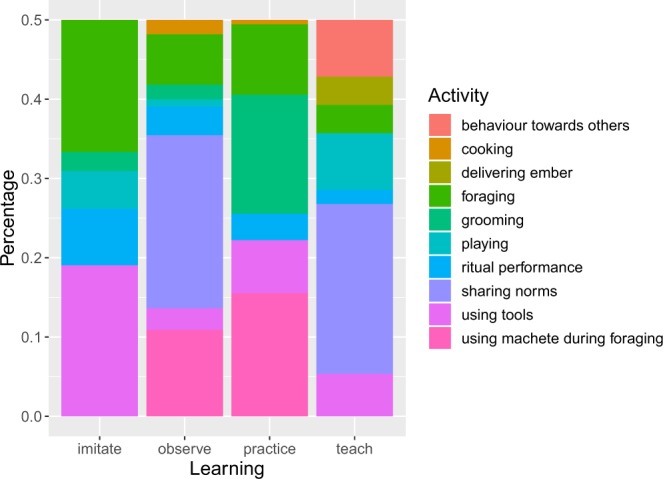
Percentage of type of activities by each learning processes. Cooking is for cooking. Delivering ember is when a focal child was asked to bring a piece of ember to the adult (learning type: teach-task assignment). Foraging comprises foraging for wild plants such as mushrooms, forest leaves and wild nuts. Grooming accounts for hair grooming. Behaviour towards others: a focal child mistreated an infant during play and received negative feedback. Playing: children’s various play activities. Ritual performance: Children’s ritual-performance play *mokondi massana*. Sharing norms: sharing of meat according to cultural norms. Using tools involve a focal child using machetes and knives. Using a machete during foraging accounts for machete use during foraging for wild yams and leaves. Note that we did not include the play-practice as a learning category here as all the play-practice events occurred during play.

Imitation, observation and practice were employed for learning visually transparent skills, whereas teaching occurred principally for the transmission of social norms. We observed that imitation, observation and practice were mainly used for foraging, grooming and cooking (Fig. 2). BaYaka children learnt foraging and tool use activities first imitating and observing older children and adults, later mostly by practicing. By middle childhood, BaYaka girls were already digging yams and using machetes efficiently. Girls started practicing foraging for wild plants by accompanying adult women in their foraging trips from early childhood. We also observed young girls going on mushroom foraging trips by themselves, where a pair of 6-year-old twins already knew which mushrooms to pick up and a younger girl observed them.

Teaching occurred in ~20% of tool use events (3 out of 14 events). Teaching, however, did not involve instructions of how to use a tool, but rather occurred when a child’s action interfered with the task of the alter. For example, in one event a 13-month-old infant was given negative feedback because she was spoiling meat while imitating an older child when cutting an animal with a knife. 41% of teaching occurred during cutting and sharing of a wild animal in two unique events, where a focal child was monitored and instructed by an adult on how to distribute meat in accordance with sharing norms. In one of these events, younger children were present, observing an adolescent boy distribute the meat whilst waiting for their share, as such meat sharing norms accounted for 44% of the observational learning (Fig. 2).

In our video sample, 9 unique play events comprised 62% of the entries (294 out of 477), some of these play activities involved teaching and imitating (Fig. 2). Half of the events where teaching occurred during play involved a young girl receiving negative feedback from older children and adults on how she treated an infant. On another occasion, an infant was moved by her aunt to teach her how to properly sit on a swing during play. The same focal child was also observed imitating older children’s actions in a playgroup. Adults can be present during children’s *mokondi massana*, but they rarely interfere. However, on one occasion a young woman instructed girls to pass a piece of clothing to the boys who were about to dress the forest spirit in the secret path, which we coded as teaching (Fig. 2).

### Boys spend more time playing than girls and the transition from play to work occurs earlier in girls

Out of 9 unique play events, 3 consisted of mixed girls’ and boys’ playgroup, two consisted of only boys, two only girls, and two consisted of a mixed group where adults were present. Our observations of girl-only playgroups came from two events, one of a 7.5-year-old girl using a can as a baby doll while her younger sister observed, and the other with three girls play-dancing a women’s ritual. In our video sample, boy-only playgroups were larger and the median age was older. For example, one boy-only group included 7 boys (median age 12.8) play-hunting. There were more boys than girls recorded in play events (12 girls and 21 boys), this effect was marginally significant (Table 1).

**Figure 3:**
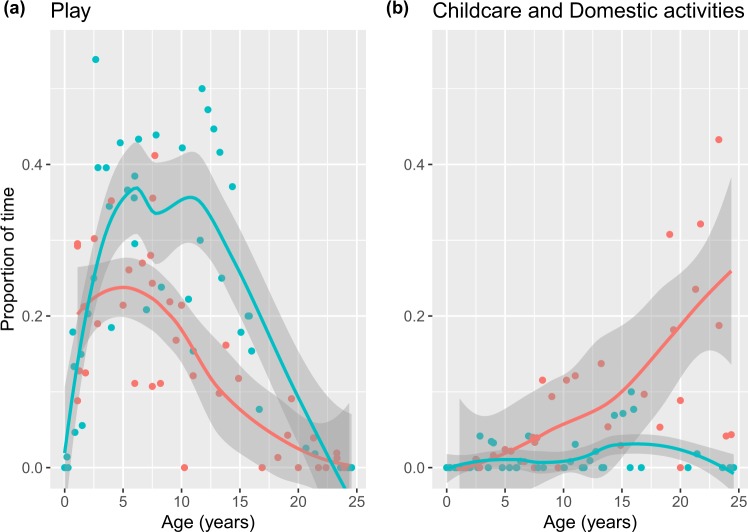
Scatterplot of proportion of in-camp time spent at (**a**) play and (**b**) childcare and domestic activities by age (years). Red denotes females, blue denotes males. Curves fitted using loess. Shaded areas represent 95% confidence interval. N: male = 45, female = 40. Note that individuals with less than 10 scan observations were excluded.

Our time allocation data also showed that BaYaka boys spent more time in play than girls (Fig. 3, Table 2). For girls, the amount of time spent playing decreased after 5 years of age when they started spending a greater amount of time working in childcare and domestic activities compared to boys (Fig. 3). In contrast the amount of time spent in play for boys remained very high (around a third of their in-camp hours) between ages 5–12 and declined continuously throughout the teenage years (Fig. 3). Whilst the decline in play occurs earlier in girls, both sexes display a similarly low level upon reaching adulthood. During infancy, there is no difference between the sexes in the amount of time spent in domestic activities and childcare. Having entered childhood girls spend more time in these activities than boys and this difference increases significantly with age (Fig. 3, Table 2).

**Table 2:** Linear regression models for the proportion of in-camp time spent at play or childcare and domestic activities.

	Model 1: Play		Model 2: Childcare and domestic activities		Model 3: Childcare and domestic activities	
	Coefficient (SE)	P value	Coefficient (SE)	P value	Coefficient (SE)	P value
Age	−0.01 (0)	<0.001	0.01 (0.00)	<0.001	0.01 (0)	<0.001
Sex (male)	0.1 (0.03)	<0.01	−0.07 (0.02)	<0.001	0.05 (0.03)	0.10
Age * Sex (male)					−0.01 (0)	<0.001
N	96		96		96	
Adjusted R-squared	0.18		0.27		0.42	

Including the *Age* * *Sex* interaction term significantly increased the model fit (for Models 2–3: P[χ^2^_1_ > 24.24] < 0.001). Coefficient is the regression coefficient obtained from the model, and SE is its standard error.

## Discussion

Our results showed that while hunter-gatherer infants learnt through imitation and observing, play became more important as children grew older. Children employed practice-based learning at all ages, but their efficiency at tasks such as using tools was low in infancy. Our video observations suggest that, once children learn the basics of a skill through observing and imitating others, they start practicing those skills from early ages. In our data, learning through being taught also occurred at all ages. Teaching was employed for the transmission of abstract information supporting the view that this form of learning has co-evolved with cumulative culture. As the skills and knowledge of our species have become increasingly sophisticated owing to their cumulative nature, a reliance on teaching likely became increasingly important to deal with their complexity and opaque nature. Observational and imitative learning, on the other hand, was used for domains that are visually transparent to observer. Our findings are in line with previous observations of the acquisition of foraging skills in hunter-gatherers: in early infancy, children accompany parents in subsistence tasks, for example by walking with or being carried by older females in the forest during foraging trips, and learn by observation, imitation and play-practicing^3,4^. From infancy to early childhood they transition into playgroups and learn by play, participation and practice^3,4,33^.

Our observations supported our prediction that children employ different social learning processes for different activity domains. Observations and imitative learning played the major role in learning skills that are non-abstract and visually transparent to the observer, such as foraging, using tools, cooking, food processing and grooming. Although teaching occurred during some events of tool use (Fig. 2), it was not to give instructions on how to use the tool, but rather for either giving a negative feedback or redirecting the infant because her actions interfered with the activity of the adult. As predicted, teaching occurred to transmit abstract information that can only be transmitted via direct instruction, for instance the cutting and distribution of meat in accordance with cultural norms around food sharing. These findings mirror ethnographic reports of the role of teaching in the acquisition of social norms in hunter-gatherers^5,39^. In her detailed analyses of *mosambo* (public speaking) events of BaYaka, Bombjaková^39^ concludes that teaching occurs during these adult speeches in the form of repeated negative or positive feedback to reinforce the importance of helping others and cooperation.

More broadly, these findings support the hypothesis that teaching co-evolved with cumulative culture to facilitate the transmission of information that cannot be transmitted with high fidelity via other social learning processes or asocial learning^18^. By using laboratory experiments on the transmission of Oldowan tool making skills, researchers have argued that tool making generated a gradual selection from gestural to verbal teaching and language in human evolution^40^. Other scholars, however, argue that gesture, rather than speech, was the principal transmission mechanism for tool-making skills, whereas speech might have evolved later, in response to complex inter and intra-group social interactions^41^. Similarly, our findings reinforce the importance of imitation, observation and practice in the learning of visually transparent tasks such as tool-use among hunter-gatherers and suggest that visual aids such as gestures and non-verbal demonstrations may have been more important in the transmission of stone tool making in early hominins. Verbal teaching and language, however, might have evolved for navigating complex social landscapes and tasks like negotiating group activities or transmitting and enforcing social norms. Our video observations on the uses of verbal teaching provides a preliminary support to this claim which should be tested in future studies. We suggest that the efficacy of transmission in social learning experiments is task-dependent. This suggestion can be tested by using experiments examining the role of different social learning processes on a range of visually transparent and more abstract social tasks.

It is noteworthy that absence of evidence is not equivalent to evidence of absence. Although, we observed no instances of direct teaching in tool-use, a recent study suggested the BaYaka and chimpanzees employ teaching for the transmission of a particular nut cracking technique where with one hand the cracker holds the nut on the sharp edge of a machete that she stabilizes between her feet and then strikes it with a wooden stick using her other hand^42^. It is a relatively complex task, since it requires the use of two tools and four limbs. Nevertheless under 15% of BaYaka *Panda* nut cracking events actually included verbal teaching, nor did teaching have any significant effect on the apprentice’s learning curve^42^, thus these results do not contradict our suggestion that verbal teaching and language likely evolved for the purposes of navigating complex social landscapes rather than visually transparent mechanical tasks.

One reason why there has been a debate on the presence of teaching in small-scale groups is that it occurs rarely and can be very subtle. Teaching does occur as shown here and elsewhere^20,25,26,42^, but unlike direct teaching observed in Western education it takes more subtle forms such as monitoring the child’s actions during a complex task like meat sharing and only intervening when necessary. In fact, a similar debate exists about the universality of sensitive responsiveness in childrearing^43^. Our video observations show that children as young as four years old are capable of noticing infants’ signals and responding to them promptly, but the interactions can be subtle^43^. Rather than talking directly to infants, caregivers often monitor infants while giving them the freedom to explore^43^. This is also the case for hunter-gatherer teaching.

Both our video observations and time allocation data revealed that BaYaka boys spent more time at play than the girls. In fact, while the time spent in play for girls declined throughout childhood, boys spent increasingly more time playing into their teens, after which we observed a decline (Fig. 3), Boyette^36^ found a similar pattern in Aka foragers. This is likely due to the emerging sexual division of labour in childhood as we observed that BaYaka girls gradually engaged in childcare and domestic activities more often than boys. Moreover, in our video recordings girls comprised almost 70% of practice events. The majority of these events concerned foraging related activities that BaYaka women do. Indeed, BaYaka girls start practicing foraging wild plants from early childhood, whereas boys only start accompanying adult men in night hunting trips during adolescence. Perhaps this is because a young unskilled hunter is more of a hindrance than an unskilled gatherer, as he alerts prey to the hunters’ presence if he is not competent at moving through the forest quickly and quietly. Boys, nevertheless, often engage in play-hunting in boy-only playgroups, where these skills can be acquired. Similar observations were also made for Baka hunter-gatherers in South-eastern Cameroon. Accordingly, sex-differences in Baka children’s subsistence activities emerge at an early age where girls engage in childcare, fishing and cooking more often than boys^44^. Recent observations on Hadza children’s activities also showed girls to spend more time in work-related economic activities in camp^37^. Thus, we suggest that the difference in time spent in play between boys and girls is a result of sexual division of labour and the play-work transition that occurs earlier for girls.

In summary, our data highlights the importance of imitative and observational learning during infancy and early childhood for the acquisition of visually transparent skills, and teaching for the transmission of abstract information related to social norms. As children grow older, play-practice replaces imitative and observational learning and boys spend more time in play than girls, who begin engaging in economic production at younger ages. Our findings help us understand how hunter-gatherer children emerge as self-sufficient, autonomous adults who have developed the necessary skills and knowledge to survive in environments where resources shift in space and are difficult to acquire, and further our understanding of the interactive relationship between human sociality, cumulative cultural evolution, teaching and language.

## Methods

### Study population and fieldwork

Mbendjele BaYaka hunter-gatherers are a subgroup of the BaYaka Pygmies whose residence spans across the rainforests of the Republic of Congo and Central African Republic. The BaYaka live with kin and nonkin in multi-family camps consisting of a number of huts in which nuclear families reside^45,46^. BaYaka subsistence techniques include hunting, trapping, fishing, gathering forest products such as wild yams and caterpillars, honey collecting and trading wild products in exchange of farmed food, cigarettes and alcohol. The level of trade and market integration vary between different groups^47^. The BaYaka, like most hunter-gatherers, are heavily reliant on cooperation, food sharing and alloparenting despite low average genetic relatedness in camp^45,48–50^. They follow specific cultural norms on how to cut and share hunted meat properly^51^.

The fieldwork took place from April to August 2014 in the Sangha and Likouala regions of Congo-Brazzaville. The first author took naturalistic video recordings of various children’s activities during daytime in three different BaYaka camps located in the forest near mud roads opened by a logging company. The recordings included both in-camp and out of camp activities. Two of the study camps (Longa, n = 60: 26 children, 34 adults, and Masia, n = 43: 24 children and 19 adults) were located in a region called Minganga, one-hour walking distance from each other (~7 km), while the other camp, Ibamba (n = 62: 27 children, 35 adults), was located ~110 km away from the other two camps, in the Ibamba region, south of Minganga. There is not constant migration between these two regions, but people may come together for a large ritual ceremony or an individual may migrate to marry. Since these regions are well isolated, different ritual ceremonies evolve in different regions^52^. In all campsites, individuals had full access to the forest and foraged daily. In Longa and Masia, people frequently traded forest products (parts of hunted meat, *koko* (*Gnetum bucholzianum*) and Marantaceae leaves) with famers, whereas in Ibamba trade was very limited as the mud road was no longer in use. People in both regions moved, in family groups, to deeper parts of the forest depending on the availability of food resources (e.g. for fishing in the dry season between December and March, and for collecting caterpillars in August) or presence of a land-related conflict with non-Pygmy groups. The BaYaka frequently visit family members and friends in nearby campsites and villages, so the composition of the camps is fluid. There was no school near any of the camps visited. The nearest school was located in a farmer village, which was about 2–2.5-hour drive from the camps Longa and Masia. Some children temporarily attended this school when their families moved to the farmer village for a period of time. The camp Ibamba was located in a more remote place with no access to school.

### Video material and sample composition

110 video recordings were analysed, totalling 5.7 hours (344 min) of continuous observation. The footage contained learning and play activities performed by children in 21 unique events. A unique event is a continuous event with the same group of people, for example a foraging trip to dig out tubers. A unique event might have been recorded in one or several videos, and a recording can contain one or more learning episodes (each one described in a different entry). The videos contained observations of a single or several different individuals. Whenever more than one child was recorded in the same video, the behaviour of each one of them was coded separately and the time of observation was added to the total time. The final sample of our analysis contained 477 entries.

The number of children recorded was 40 (18 girls and 22 boys). For 34 children, we had age estimates using a statistical method for calculating age based on relative age rankings^53^. The youngest participant was 1.1 years old (13 months) and the oldest participant was 14.4 years old. The mean and median ages for participants were 8.2 and 8, respectively. The median age for all social learning entries (including repeated entries of the same focal child) was 7.7. Following the literature on the developmental stages of human childhood, we used the age groups shown in Table 3 for infancy, early and middle childhood, and adolescence^54,55^.

**Table 3:** Video sample composition.

Age (years)	Developmental stage	Girls	Boys	Number of entries
1–4	Infancy to early childhood	4	1	63
4–7	Early childhood	5	3	93
7–13	Middle childhood	7	9	242
13–16	Adolescence	2	3	53
Participants without age estimate			6	26
Total		18	22	477

### Coding

The videos were initially watched and coded by Salali. Salali used Hewlett’s coding scheme for teaching events^20^, but extended the scheme for other learning behaviours to include imitative learning, observation, practice and play-practice (Table 4). Bouer watched and coded the videos independently based on the coding scheme used by Salali. There was 98% inter-coder reliability (only 9 out of 477 were coded differently, Cohen’s Kappa for the two coders was 0.97 (*p < *0.001)). The authors discussed these differences and reached a consensus.

**Table 4:** Processes of social learning coded.

Type of learning	Description	Number of entries	Number of focal children
Imitate	The focal child is imitating a model doing a specific task or activity such as taking the kernels out of a wild plant.	21	4 (3 girls, 1 boy)
Observe	The focal child is observing a model who engages in an activity that requires specific knowledge or skill such as digging a wild tuber or sharing a hunted meat.	55	16 (13 girls, 3 boys)
Play-practice	The focal child is in a playgroup practicing a skill or acquiring cultural knowledge through play. For example, boys are play-hunting or a mixed children group is performing a forest spirit ritual through play.	283	33 (12 girls, 21 boys)
Practice	The focal child is practicing a skill in the absence of a model. We coded this behaviour as practice as opposed to trial and error, because trial and error indicates individual learning, whereas practice may include those skills that were previously acquired by imitating and observing others.	91	16 (11 girls, 5 boys)
Teach: move body	The model moves the focal child’s body to show her/him what (not) to do.	2	6 (3 girls, 3 boys)
Teach: task assignment	The model gives the focal child a task.	5	
Teach: negative feedback	The model makes displeasing comments or sounds.	7	
Teach: verbal	The model gives verbal instructions.	7	
Teach: redirect	The model redirects the focal child to another location or activity because s/he does something inappropriate.	2	
Teach: opportunity scaffolding	The model gives the focal child a learning opportunity by providing a tool and monitoring the activity.	4	

In addition to the type of social learning, each entry contained information on the type of activity the focal child engaged in. The video sample comprised the following activities: cooking, meat sharing, foraging for wild mushrooms, wild yams and plants, grooming (a common social activity in the BaYaka to remove hair lice from hair and/or promote social bonding), tool use (machetes and knives), using a machete for digging yams (coded as tool use during foraging), and an activity when a focal child is asked to bring something to an adult. In addition to these activities, we coded play events. These events included boys play-hunting where a group of boys used hollow sticks as pipes to hunt an insect; jumping on *Megaphrynium macrostachyum* leaves that were collected by women and girls to be sold; walking on wooden sticks; boys playing a ball game with sticks; girls playing with baby dolls made of empty cans; swinging; and children’s ritual-performance play *mokondi massana*. If teaching or imitative learning occurred during the play activities, we coded those as the learning processes. Otherwise, we coded the learning process occurred during a play event as “play-practice”.

### Activity budgets

In addition to the video recordings, we used data from scan sampling to examine BaYaka children’s time spent in play. The scan data recorded which activities each individual was engaged in at systematic ‘snapshots’ in time. Scans were taken at the same camps where video recordings were taken. Scan data was collected hourly throughout the day between 6:00 and 18:30 and involved everyone present at the camp at the time of data collection. The recorded activities included resting, cooking, domestic activities (cleaning, collecting firewood and water, manufacturing baskets and mats, making a hut), childcare, and social activities (talking, playing, grooming, trading, singing). The total sample analysed in this study was comprised of 96 individuals under 25 years-old. On average, there were 59 scans per person. The data contained 5635 person scans in total.

### Ethics approval

This research was approved by UCL Ethics Committee (UCL Ethics code 3086/003) and the methods were carried out in accordance with the approved guidelines. Informed consent was obtained from all participants, and parents signed the informed consents for their children (after group and individual consultation and explanation of the research objectives in the indigenous language). Research permission was granted by the Republic of Congo’s Ministry of Scientific Research.

### Statistical analyses

The major limitation of our methods was that our video recordings were opportunistic, not systematic. As a result, participants were not equally represented in our sample. We controlled for the repeated measures by using mixed effects models. We conducted separate regression analyses for each learning process (imitation, observation, practice, play and teach). Our response variable was the presence/absence of a learning process, and the predictor variables were: age and sex of the focal child (fixed effects) and the focal child’s ID (random effect).

We calculated the median ages for each learning process by considering how represented each child is in the dataset. For example, if we had two imitative learning entries for a focal child aged 2, and one entry for a focal child aged 3, the median age for this learning process was calculated as 2.

We calculated the proportion of time spent in different activities by measuring the percentages of scans in which the focal child was observed engaging in a specific activity (e.g. play, childcare and domestic activity) with respect to the total number of scans for the child on any particular day. We used multiple regression analysis to examine the relationship between age, sex (predictors) and the proportion of in-camp time spent in play, childcare and domestic activities (response variable).

## Data Availability

The datasets generated during and/or analysed during the current study are available from the corresponding author on reasonable request.
